# Preparedness of Health Care Professionals for Delivering Sexual and Reproductive Health Care to Refugee and Migrant Women: A Mixed Methods Study

**DOI:** 10.3390/ijerph15010174

**Published:** 2018-01-22

**Authors:** Zelalem B. Mengesha, Janette Perz, Tinashe Dune, Jane Ussher

**Affiliations:** 1 Translational Health Research Institute (THRI), School of Medicine, Western Sydney University, Penrith, NSW 2751, Australia; J.perz@westernsydney.edu.au (J.P.); t.dune@westernsydney.edu.au (T.D.); J.ussher@westernsydney.edu.au (J.U.); 2 School of Science and Health, Western Sydney University, Penrith, NSW 2751, Australia

**Keywords:** refugee and migrant women, sexual and reproductive health, training, knowledge, confidence, health care professionals

## Abstract

Past research suggests that factors related to health care professionals’ (HCPs) knowledge, training and competency can contribute to the underutilisation of sexual and reproductive health (SRH) care by refugee and migrant women. The aim of this study was to examine the perceived preparedness of HCPs in relation to their knowledge, confidence and training needs when it comes to consulting refugee and migrant women seeking SRH care in Australia. A sequential mixed methods design, comprising an online survey with 79 HCPs (45.6% nurses, 30.3% general practitioners (GPs), 16.5% health promotion officers, and 7.6% allied health professionals) and semi-structured interviews with 21 HCPs, was utilised. HCPs recognised refugee and migrant women’s SRH as a complex issue that requires unique skills for the delivery of optimal care. However, they reported a lack of training (59.4% of nurses, 50% of GPs, and 38.6% of health promotion officers) and knowledge (27.8% of nurses, 20.8% of GPs, and 30.8% of health promotion officers) in addressing refugee and migrant women’s SRH. The majority of participants (88.9% of nurses, 75% of GPs, and 76% of health promotion officers) demonstrated willingness to engage with further training in refugee and migrant women’s SRH. The implications of the findings are argued regarding the need to train HCPs in culturally sensitive care and include the SRH of refugee and migrant women in university and professional development curricula in meeting the needs of this growing and vulnerable group of women.

## 1. Introduction

Health care professional (HCP) interactions with patients from refugee and migrant backgrounds are increasing, due to growing diversity in migration to countries such as Australia [1]. In addition to clinical training and knowledge, caring for refugee and migrant patients requires an understanding of factors in the broader socio-ecological environment that impact the level and type of care to be provided, such as migration circumstances, cultural backgrounds and language needs [2]. Previous research suggests that cross-cultural training increases HCPs’ interest in seeing migrant patients [3]. Furthermore, HCPs who received cultural competency training were more likely to use professional interpreters to improve communication and understanding [4,5] and to make changes in their practices to accommodate migrant clients [5]. The HCP’s cultural awareness also improves communication, understanding and treatment compliance in consultations with people from refugee and migrant backgrounds [6]. Despite having these benefits, there is a growing concern in migrant resettlement countries that HCP education is not responding to the changing demographics of the population, with many HCPs not satisfied with their knowledge and understanding in providing appropriate health care to patients from refugee and migrant backgrounds [2,7,8].

There are several issues particular to refugee and migrant women that have bearing on their interpersonal interactions with HCPs within a SRH context. For example, some refugee and migrant women have experienced rape and other forms of sexual abuse before and after resettlement in host countries [9,10]. Consequently, they may experience SRH and psycho-social problems [11], such as unsafe abortion, sexually transmitted diseases, including HIV, and trauma [12]. Furthermore, talking about sex and menstruation is taboo among many women from refugee and migrant backgrounds, which may present a challenge for HCPs who are trying to understand the women’s needs during consultations [13,14]. Some refugee and migrant women also attend SRH consultations with their husbands which pose challenges for HCPs to provide care as male partners commonly dominate the consultation leaving women unable to disclose their SRH needs in the presence of their partners [15]. This suggests that HCPs require skills in broaching SRH and navigating patient relationships in ways that allow them to satisfy refugee and migrant women’s SRH needs. Without this acuity, the level of care that HCPs are able to offer to these women is reduced [16].

A number of studies in Australia have examined the provision of cross-cultural training to HCPs who have day-to-day interactions with people from refugee and migrant backgrounds. For example, Watt and colleagues report that only 23.8% of General Practice (GP) registrars received cross-cultural training, despite more than half of their patients coming from a cultural background different from the provider [17]. In another study, GPs reported acquiring cross-cultural knowledge and competency mainly through exposure to patients, as opposed to formal training [18]. These studies did not examine the type of health care provision, or the range of migrant populations seen by GPs or other health care providers. A comprehensive socio-ecological analysis of HCP’s perceptions and experiences regarding their multi-cultural training and competency is lacking.

Research into the provision of SRH care to refugee and migrant women in countries of resettlement is growing [19]. This research has concentrated on understanding the experiences of HCPs regarding language barriers and interpreter use [20], communication [21,22], expectations around language services, cultural competency and type of care [23], structural barriers to providing care [24], and the women’s familiarity with services [22]. One of the remaining gaps in knowledge is the perceived preparedness of HCPs in relation to their training, knowledge and confidence when it comes to consulting refugee and migrant women seeking SRH care. The aim of this mixed-methods study was to address this gap, in order to: (1) assess the perceived knowledge and confidence of HCPs in their ability to work with refugee and migrant women seeking SRH care; and (2) examine HCP’s training experiences and needs with respect to the provision of SRH care to refugee and migrant women in Australia.

### Theoretical Approach

Health care provision requires the involvement and coordination of several micro systems in the broader health system [25]. As such, a systematic approach which considers influences beyond the individual level is relevant to have a broader understanding of factors that impact health care access and utilisation [25]. The socio-ecological model is most central to this process as it recognises multiple domains of influence in an individual’s social environment that impact health care access and provision [26,27]. With this in mind, the socio-ecological model was selected as a framework to systematically analyse factors that influence HCP’s preparedness for providing SRH care to refugee and migrant women in Australia at four levels: Individual, Interpersonal, Organisational, and Societal levels. The model is also appropriate for mixed methods research. The quantitative component in this study considers the individual level factors while the qualitative data further explores these factors across the four levels of the socio-ecological framework. The important conceptualisation is that, as the four levels of the socio-ecological model are interconnected, so too are quantitative and qualitative data collected to examine the HCP’s preparedness to provide SRH care [28]. 

## 2. Methods

This sequential mixed methods study involved the collection, analysis and interpretation of quantitative and qualitative data to achieve the research objectives [29]. An online survey was conducted with a diverse group of HCPs to assess their knowledge, confidence, training needs and experience in relation to the provision of SRH care to refugee and migrant women. Semi-structured interviews were then conducted to explain and elaborate upon issues identified within the survey [30]. Ethical approval was obtained from the Human Research Ethics Committee at Western Sydney University (Approval Number H11034) and informed consent was obtained from all individual participants included in the study.

### 2.1. Participants 

Seventy-nine HCPs participated in this research. HCPs were a convenient sample recruited through advertisements in nursing and public health professional association newsletters and email lists, family planning clinics, and snowball sampling. To advertise the study, a leaflet which contained information about the proposed study and the opportunity to participate in a survey and semi-structured interviews was used. Table 1 presents the socio-demographic and work experience profile of the HCPs who responded to the survey. The vast majority were women (96.2%) with a mean age of 47.1 years (+12.12). HCPs included nurses (45.6%), GPs (30.3%), health promotion officers (16.5%) and allied health professionals (7.6%). They had an average work experience of 11.2 years in the health sector ranging between 2 and 41 years. The majority (58.2%) reported seeing 1–5 women daily from refugee and migrant backgrounds in their practices. HCPs reported that SRH services that refugee and migrant women most commonly sought were: contraception (64.56%), gynaecological concerns (60.46%), pregnancy related (41.77%) and infertility (40.51%). These experiences were widely distributed across women from several countries which included Afghanistan (41.33%), Sudan (40%) and Iran (40%). 

### 2.2. Procedure

#### 2.2.1. Survey

HCPs completed the survey online which consisted of 24 closed and open-ended items. The survey covered many issues which include: knowledge and confidence to provide SRH care to refugee and migrant women; past training experiences and current needs in relation to refugee and migrant women’s SRH; and barriers to the SRH care of these women. In addition, demographic questions (i.e., gender, age, years of experience, first language, country of birth, country where they received their professional training and approximate number of refugee and migrant women seen in their practice) were also included.

#### 2.2.2. Semi-Structured Interviews

After the HCPs completed the online survey they were asked to participate in a follow up interview about their experiences of providing SRH care to refugee and migrant women. Of the 79 survey participants, 32 responded positively to the invitation. Then, 21 were purposefully selected and interviewed. These participants included nurses (8), GP (5), health promotion officers (5), sexual therapists (2) and a midwife. The HCPs interviewed had an average work experience of 21 years across the public (5), private (2), public/private (4) and non-profit/NGO (10) sectors, with work experience ranging from 2 and 41 years. Individual semi-structured interviews were conducted by the first author via telephone and one face-to-face. Topics included the HCP’s perceptions of refugee and migrant women as well as their knowledge, competencies, training needs, experiences, and preferences; their use of interpreters; perceived support needs of HCPs and refugee and migrant women to facilitate access to care. The interviews were audio-recorded and lasted an average of 50 min. All the interviews were professionally transcribed with subsequent integrity checking undertaken for accuracy. To enhance clarity and readability of the results, irrelevant sentences and words were deleted and replaced by sentence ellipses (“…”). For longer quotes, pseudonyms, profession and years of work experience are also given.

### 2.3. Operational Definitions 

To better explore the perspectives of HCPs operationalising what is meant by various, and overlapping, terms is helpful. As such, in this paper the following definitions were used.

**Preparedness**: HCP’s perceived ability to provide SRH care to refugee and migrant women where having appropriate knowledge, training and confidence is perceived as being “prepared”.

**Knowledge**: the perceived facts, information, and skills acquired through experience or education that enable HCPs provide SRH care to refugee and migrant women [31].

**Confidence**: the perceived feeling or belief that one can provide SRH care to women from refugee and migrant backgrounds [32].

### 2.4. Analysis

Descriptive statistical analyses were employed to evaluate quantitative data such as demographic variables, training history, current needs and experiences and perceived confidence for providing SRH care to refugee and migrant women. Analyses were run using SPSS 24 Statistical Software (IBM Corporation, New York, NY, USA). Chi-square tests were conducted to test for differences between the professional groups in relation to their knowledge, confidence, training needs and experience.

Thematic analysis according to Braun and Clarke [33] was used to analyse semi-structured interviews and open-ended survey responses. This process started with the first author reading and re-reading the interview transcripts resulting in the identification of first order codes such as “HCP’s deficiencies”, “Strengthening GP service”, and “Training needs”. The other authors also read the transcriptions and collectively contributed to the development of the coding frame. The entire data set was then coded using NVivo, a qualitative data management and organisation software. Through attentive reading and examination of the coded data, the codes were grouped into preliminary themes. This was followed by the development of conceptual themes such as “Knowledge and confidence to provide SRH care” and “Training needs and experiences in the SRH care of refugee and migrant women” through a process which involved examining patterns, similarities and differences across the codes and preliminary themes by all the authors. Finally, the results of the survey and qualitative analyses were combined through a process of triangulation that enabled the authors to connect and interpret both data sets simultaneously through convergence and corroboration [29]. The socio-ecological model which was introduced after data analysis to make sense of the results in the broader framework helped us to identify factors that impact the HCP’s preparedness for providing SRH care to refugee and migrant women across the four levels.

## 3. Results

**Figure 1:**
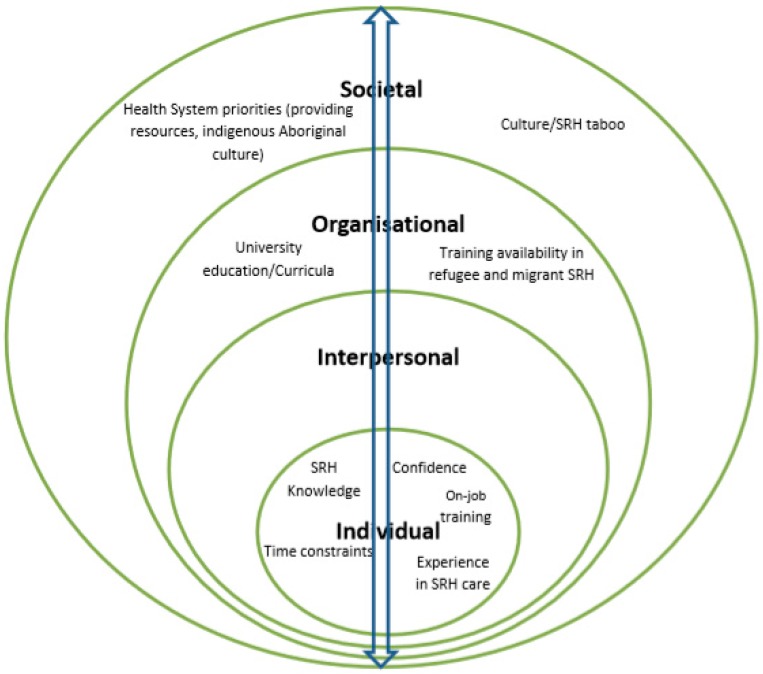
A socio-ecological analysis of factors that influence provider preparedness for delivering SRH care to refugee and migrant women in Australia.

The results are presented thematically, addressing the aims of the study: (1) knowledge and confidence: “Providers don’t want to deal with it or they can’t deal with it”; and (2) training experience and needs: “It’s an entirely different topic not covered in the universities”. Within each theme, a number of factors were identified at individual, organisational and societal levels of the socio-ecological model. These factors interact across levels to influence HCP’s preparedness for providing SRH care to refugee and migrant women. For example, at individual level the data indicated that work experience in SRH care, cross-cultural knowledge, on job training and confidence to initiate and discuss SRH influenced HCP’s ability to provide SRH care to refugee and migrant women. At the organisational level, university education/curricula and training availability in refugee and migrant women’s SRH care impacted HCP’s knowledge and confidence in SRH care provision. Influential societal level factors included SRH taboo, health system priorities of providing resources instead of training in SRH care, and emphasis on aboriginal cultural training. These suggest that HCP preparedness to provide SRH care to refugee and migrant women is influenced by multiple factors across the socio-ecological model, and multiple and multilevel interventions are necessary. Figure 1 shows a summary of factors at each level. In the presentation of the two major themes below, the ways in which the identified factors across the socio-ecological model were constructed to influence HCP’s preparedness to provide SRH care to refugee and migrant women are discussed.

### 3.1. Knowledge and Confidence: “Providers Don’t Want to Deal with It or They Can’t Deal with It”

Although no significant differences were observed between professional groups in relation to perceived knowledge and confidence, more than a quarter (26%) of HCPs rated their knowledge of refugee and migrant women’s SRH as very low or low, and 16.4% of HCPs rated their confidence to provide SRH care as low or very low (Table 2). A number of areas where HCPs across all occupational groups described their knowledge and confidence to provide SRH care to refugee and migrant women as inadequate were also identified from the semi-structured interviews. For example, many HCPs explained that engaging in SRH conversations was difficult:

Broaching the subject is the challenge I face because lots of women tell you “oh, I don’t want to talk about it.” So it’s a challenge to be able to break that barrier to let them know that it’s important to talk about what’s going on because you need to help them in regards to not having any problem now or in the future.

(Alice, GP, 35)

This perceived difficulty demonstrates the challenges and cultural barriers on which HCPs need more training to effectively raise discussions about SRH, as Hannah (GP, 31) indicated:

Hell, the majority of them (HCPs) can’t do (talk about) sexual and reproductive health to these women…You can’t sit there and giggle and say, “oh I’m sorry I’ve got to ask you a personal question.” The challenge is getting them (the women) to bring it up as they are not going to say “I have got a discharge.”

This may hinder the HCP’s ability to understand the women’s needs and provide appropriate SRH care. According to some of the HCPs, the difficulty in discussing sexual health was related to their lack of awareness about the women’s attitude towards sexual health: “I don’t know their attitude towards sexual pleasure or towards sexual practice to talk about it” (Emma, sex therapist, 6). Consequently, some HCPs may just refer refugee and migrant women to specialist services to avoid the difficulty: “Often GPs will just refer (women) to sexual health (clinics) because they don’t want to deal with it (difficulty of raising SRH discussions) or they can’t deal with it” (Amy, nurse, 17). This implies that HCPs may miss opportunities to gain experience in SRH care provision to refugee and migrant women.

Not all HCPs described themselves as inadequately prepared to discuss SRH with refugee and migrant women. Few nurses indicated that they were confident talking to refugee and migrant women about SRH, with their confidence stemming from previous training in SRH care provision: “I’ve done a number of courses as part of my work and I’m very comfortable talking about sexuality” (Alex, nurse, 42). Others reported that their confidence stemmed from their extensive work experience in SRH:

“I have worked in sexual health for a long time. So to me it’s no different to addressing the health of their eyes or their lungs or their feet or anything else. There is no cultural group that I can’t discuss these (sexual health) issues with.”

(Amy, nurse, 17)

This indicates that extended work experience in SRH care improves the HCP’s confidence to initiate and discuss SRH topics with women from refugee and migrant backgrounds. A number of HCPs shared strategies they had found effective in helping to overcome the difficulty of initiating SRH discussions with refugee and migrant women. They emphasised the need to prepare the women, by starting the discussion by highlighting the benefits of engaging in an open conversation with HCPs:

“Generally no one likes talking about that area of their health. So the way I do it is prepare them first, that I would like to ask them these questions, and give them a benefit to answering those questions for themselves. So for example, I ask this for your health. I want to make sure you’re all right. There is a positive in it for them.”

(Amy, nurse, 17)

This result highlights the importance of how HCPs initiate SRH discussions with refugee and migrant women seeking SRH care. It is imperative that HCPs use different approaches to discuss SRH as societal taboos associated with this topic may hinder the women from seeking information and service leading to misconceptions and knowledge gaps.

### 3.2. Training Experiences and Needs: Moving across Levels of Influence

HCPs in this study recognised refugee and migrant women’s SRH as a “more complex” and “entirely different topic”. They also reported that SRH care provision to refugee and migrant women requires a “different skill set to give these messages to these ladies”. Despite these acknowledgements, the majority of nurses (59.4%), 50% of GPs and 38.6% of health promotion officers had not undertaken any training or professional development that specifically addressed refugee and migrant women’s SRH (Table 2). The absence of HCP training in refugee and migrant women’s SRH was also identified by many participants across all professional groups in the interviews. HCPs provided many reasons why they had not received training in refugee and migrant women’s SRH. Some participants described refugee and migrant women’s SRH as “not covered in the universities” and that HCPs are “not taught as much as (they) could be during training”. For example, Grace (nurse, 13) explained that “there is more emphasis on providing resources than educating HCPs about refugee and migrant health issues” in the health system. Another nurse stated that “something that’s easily accessible and affordable education about these cultural issues is not available for migrant and refugee groups” (Harper, 19). Where cross-cultural training exists in the healthcare system, some HCPs reported that it focused on Indigenous Aboriginal health with little attention to refugee and migrant health:

“There is now a huge push for us to learn about the Aboriginal culture and it’s become mandatory that we do some of the training on that. But there’s nothing really to—along similar lines to educate us about these other cultures and about some of the traumas and such that people go through.”

(Grace, nurse, 13)

Whilst some of the HCPs did not mind having more training on the SRH of refugee and migrant women, others reported a lack of time to undertake additional training:

“So time constraint is a big issue for health professionals. Because they’re always short of time, I think that they don’t spend the time or don’t put the time aside to do extra training in terms of sexual and reproductive health care, and when training does come up, often they can’t go because they’re so short of time, or because they just don’t want to do it because it’s too hard.”

(Amy, nurse, 17)

Consequently, many HCPs noticed that they were underprepared to provide SRH care to refugee and migrant women: “We’re taught some and we’re taught not enough in so many areas (of refugee and migrant women’s SRH). There are a lot of areas that I feel inadequate in” (Hannah, GP, 31). Finally, several HCPs suggested areas where additional training is needed. For instance, Chloe (nurse, 13) explained that with “cultural awareness training … I like to know what messages I’m conveying subconsciously. I would like to improve that and be able to convey my messages a bit more clearly”. Importantly knowing the women’s cultural background is reported to be important in delivering SRH messages. Grace (GP, 13) added, “We need more education on the cultures, cultural norms and the cultural expectations of these migrant and refugee women and some understanding of how they would like care to be provided when it’s care around sexuality and sexual health”. Learning about the role of culture and ethnicity in refugee and migrant women’s SRH was viewed by many as facilitating self-reflection and analysis and leading to better patient care:

“Sometimes you can be having a consultation—it’s all going well, and then something just changes. I think I would really like to know why in some of those cases. Some of them you can sit back and think, oh, I shouldn’t have said that or I should have said this differently, or I should have backed off and asked that in a different way. I think having increased knowledge helps to do that sort of analysis afterwards.”

(Chloe, nurse, 13)

HCPs described increased SRH knowledge as an imperative to improved service delivery versus being elective with regards to professional development. Tayla (GP, 22) noted, “All GPs (need) to be adequately trained in sexual and reproductive health, rather than regarding it as an optional extra for those with special interest”. Few participants also recommended training to medical support staff such as receptionists to help them understand the contexts of refugee and migrant women and assist the women in booking and re-booking appointments:

“I guess that’s the same for even potentially medical receptionists as well, because if a woman misses her appointments a couple of times, or comes extremely late for an appointment, sometimes that can be quite frustrating for receptionists who have to re-book their appointments, yet they don’t actually understand the reasons why that might be occurring.”

(Kokob, health promotion officer, 8)

Given that 88.9% of nurses, 75% of GPs and 76% of health promotion officers reported need for and demonstrated willingness to engage with further training in this area, the modes of delivering for such training are important. The majority of the HCPs preferred online self-directed learning (66.67%), workshop methodologies (65.22%) and professionally accredited courses (43.48%). These results imply that HCP SRH education programs in the health system need to consider their audience, scope, timing, content, accessibility and recognition relating to modes of training delivery.

## 4. Discussion 

Previous research has recommended the need to prepare the health workforce and the health system to effectively respond to the changing demography in major refugee and migrant resettlement countries such as Australia [34,35,36]. Whilst there have been efforts in the health system to improve cultural competency for healthier living and environments [37,38], less effort has addressed the preparedness of HCPs in relation to their knowledge, confidence and training to provide SRH care to referee and migrant women.

HCPs in this study reported a lack of knowledge regarding refugee and migrant women’s SRH issues that may impact the quality of care they are able to offer these women [16]. Similarly, HCPs in other multicultural societies reported that their undergraduate and postgraduate studies had not prepared them to provide care for women from refugee and migrant backgrounds [39]. The finding that HCPs who received training in refugee and migrant women’s SRH were comfortable with their knowledge and confidence suggests that further training would likely result in improved knowledge and competency in this area [2,5]. This implies the need to improve cultural competency training in higher education institutions and training in culture, sexuality and health and core concepts of working with refugee and migrant women as they are pertinent to the care of these women in SRH [40]. The SRH issues of refugee and migrant women should also be incorporated in higher education and other professional development curricula for HCPs. 

Another important finding at individual level was the enthusiasm of the majority of HCPs to undertake further training/professional development activities in refugee and migrant women’s SRH. This finding highlights the key role that HCPs can take in informing the development of effective and relevant cross-cultural training in SRH [5]. The areas identified for additional training included cultural sensitivity and awareness and health issues specific to refugees and migrant women. The training needs expressed by the HCPs can be associated with the perceived challenges of providing SRH care to refugee and migrant women [4]. For instance, majority of the HCPs in this study reported having difficulties in effectively initiating and discussing SRH with refugee and migrant women due to societal taboos associated with this topic. This may mean that the SRH needs of these women are to some extent under addressed [41]. Past research suggests training of HCPs working in cross-cultural care to improve interpersonal communication and understanding with patients from refugee and migrant backgrounds [42]. 

This study has a number of strengths and limitations. The sequential mixed methods design was strength as it helped us to better explain and elaborate issues raised in relation to the HCP’s knowledge, confidence and training to provide SRH care to refugee and migrant women. A limitation was that HCPs may have provided socially desirable responses as they self-rated their knowledge, training and confidence to provide SRH care to refugee and migrant women. As such, the HCP’s knowledge, confidence and training may have been inflated. Due to response bias, HCPs who participated in this study might also be those who were more interested in refugee and migrant women’s SRH. Majority of the HCPs were also women which makes it difficult to ascertain if provider gender influences their preparedness. As a result, generalisation of the findings to all HCPs in Australia could not be drawn. Further research on how refugee and migrant health and SRH is being taught in universities and professional development courses; whether it is integrated into the curriculum; and how students feel about their knowledge and skills to provide care to women from refugee and backgrounds is recommended. 

## 5. Conclusions 

This study has provided evidence that a significant proportion of HCPs in Australia may not be providing optimal SRH care to refugee and migrant women even though many put efforts to accommodate them. This is mainly attributed to the broader interplay of influencing factors at individual, organisational and system levels suggesting the need for multilevel strategies to improve the provision of SRH care to refugee and migrant women. The policy implications of the findings are argued regarding the need to train HCPs in culturally sensitive care and include the SRH of refugee and migrant women in university and professional development curricula. These interventions are vital to the provision of gender-sensitive and culturally appropriate SRH care to this growing and vulnerable group of Australia’s population [11,13,43].

## Figures and Tables

**Table 1:** Socio-demographic and work experience characteristics.

Characteristic		Frequency (*n*)	Percentage (%)
Gender	Women	76	96.2
	Men	3	3.8
Occupation	Nurse/Midwife	36	45.6
	GP	24	30.3
	Health promotion officer *	13	16.5
	Allied health professionals **	6	7.6
Work experience in years	1–10	33	41.8
	11–20	25	31.6
	21 and above	21	26.6
Refugee and migrant women seen daily	0	27	34.1
	1–5	46	58.2
	>6	6	7.7
SRH services refugee and migrant women commonly accessed	Contraception	51	64.56
	Pregnancy related (Antenatal care, delivery and postnatal care)	33	41.77
	Abortion	29	36.71
	Sexually transmitted infections (Information, screening and treatment)	29	36.71
	Screening (Chlamydia and Cervical cytology)	36	45.57
	Infertility	32	40.51
	Safer sex options	17	21.52
	Sexual pain and discomfort	30	37.97
	Sexual violence and unwanted sex	17	21.52
Background of women seen	Afghanistan	31	41.33
	Iran	30	40
	Sudan	30	40
	Iraq	29	38.67
	Myanmar/Burma	19	25.33
	Somalia	18	24
	Bhutan	6	8
	Congo (DRC)	6	8
	Others ***	36	48

* Health promotion officer includes bilingual health educators and health educator managers. ** Allied health professionals include psychologists and sex therapists. *** Others include South East Asia, Zimbabwe, Turkey, Saudi Arabia, and Egypt.

**Table 2:** HCP’s knowledge, confidence, training needs and experience with refugee and migrant women’s SRH care.

Variable		Occupation			Test of Group Difference	
		Nurse	GP	HPO	χ^2^	*p*
Knowledge	Very low/low	10 (27.8%)	5 (20.8%)	4 (30.8%)	2.04	0.73
	Moderate	15 (41.7%)	13 (54.2%)	4 (30.8%)		
	High	11 (30.6%)	6 (25.0%)	5 (30.8%)		
Confidence	Very low/low	7 (19.4%)	3 (12.5%)	2 (15.4%)	1.95	0.74
	Moderate	18 (50.0%)	14 (58.3%)	5 (38.5%)		
	High	11 (30.6%)	7 (29.2%)	6 (46.2%)		
Previous training	Yes	11 (30.6%)	12 (50%)	8 (61.5%)	4.58	0.10
	No	25 (69.4%)	12 (50%)	5 (38.5%)		
Need for further training	Yes	32 (88.9%)	18 (75%)	10 (76.9%)	2.04	0.33
	No	4 (11.1%)	6 (25%)	23.1%)		
